# Cervical Cancer Prevention in Racially Disparate Rural Populations

**DOI:** 10.3390/medicines6030093

**Published:** 2019-09-04

**Authors:** Patti Olusola, Kia Ousley, Harrison Ndetan, Karan P. Singh, Hirendra Nath Banerjee, Santanu Dasgupta

**Affiliations:** 1 Department of Family Medicine, The University of Texas Health Science Center at Tyler, Tyler, TX 75708, USA (P.O.) (K.O.); 2 Department of Epidemiology and Biostatistics, The University of Texas Health Science Center at Tyler, Tyler, TX 75708, USA (H.N.) (K.P.S.); 3 Department of Natural, Pharmacy and Health Sciences, Elizabeth City State University, Elizabeth City, NC 27909, USA; 4 Department of Medicine, The University of Texas Health Science Center at Tyler, Tyler, TX 75708, USA

**Keywords:** cervical cancer, HPV, Pap test, colposcopy

## Abstract

**Background:** Undergoing a timely Pap smear, high-risk human papilloma virus (HPV)- and colposcopy-based testing can reduce HPV-associated cervical cancer (CC) development in women. However, in rural areas, women and minorities without insurance do not undergo periodic assessment and remain at greater risk of HPV infection and CC. **Methods:** In this study, 173 women from rural East Texas with various ethnic backgrounds were examined thorough HPV/Pap-based testing and colposcopic assessment. **Results:** Of the 113 informative cases, 77% (87/113) were positive for high-risk HPV infection and 23% of subjects (26/113) were negative. Associations between HPV positivity with young age (*p* = 0.002), and a low number of pregnancy (*p* = 0.004) and births (*p* = 0.005) were evident. Women with long-term use of contraceptives (OR 1.93, 95% CI, 0.80–4.69) were associated with increased risk of HPV infection. African-American women had a higher risk of abnormal Pap outcome compared to Caucasians (OR 5.31, 95% CI, 0.67–42.0). HPV seemed to be a predictor of abnormal Pap outcome (OR 1.77, 95% CI, 0.48–6.44) in these subjects. Unmarried/widowed/divorced women had an increased abnormal Pap test outcome compared to married women or women living with a partner (*p* = 0.01), with over 278% increased odds (OR 3.78 at 95% CI, 1.29–11.10). Insured women undergoing periodic checkups were detected early with high-risk HPV infection and abnormal Pap test/colposcopic outcome. **Conclusions:** Comprehensive and timely screening of uninsured women and minorities in rural East Texas are warranted, which could potentially prevent the onset of HPV-associated CC.

## 1. Introduction 

Cervical cancer (CC) is one of the leading causes of death among women [1,2,3,4,5,6,7]. In the United States, 13,170 new cases of CC are expected to be diagnosed with an estimated death of 4250 women in 2019 [3,4]. In the State of Texas, an estimated 1363 women are expected to be diagnosed and 431 are expected to die from the disease [3,4]. The frequency of CC incidence is higher in African-American and Hispanic women compared to Caucasian American women [5]. The major risk factors associated with CC development include high-risk human papilloma virus (hrHPV) infection, use of oral contraception, sexual promiscuity, cigarette smoking, childbirth, marital status and age [3,4,5,6]. Among these various risk factors, persistent infection with hrHPV appears to be the major driver of CC development [1,2,3,4,5,6]. To date, 216 subtypes of HPV have been discovered [7]. In the early stages, hrHPV-associated CC development is asymptomatic [8,9]. If not screened, high-risk HPVs may remain undetected and result in oncogenic transformation leading to CC in the later stages of life, thereby warranting periodic checkups of populations at risk [8,9]. In rural areas, women with insufficient or no health insurance and from various ethnic backgrounds are less likely to be examined regularly for HPV and associated CC risk assessment. Thus, these medically underserved populations remain at greater risk of developing CC in their lifetime [3,9,10]. Preventative and strategic screening of these women is thus of paramount importance to reduce CC incidence and associated mortality [5,9,10,11,12]. 

The present study has undertaken an initiative to address this problem and evaluated outcomes from 173 women in rural areas of East Texas for CC risk determination through hrHPV detection, Pap test and colposcopy-based examination. Through this evaluation, we have observed an association between HPV positivity and increased risk with young age, low number of pregnancies and births and long-term contraceptives use. Unmarried/widowed/divorced women had an increased abnormal Pap test outcome compared to married women or women living with a partner. On the other hand, African-American women exhibited higher risk of abnormal Pap outcome compared to Caucasian American women. 

## 2. Materials and Methods 

### 2.1. Patient Population and Ethical Statement 

Tyler is located in East-Central Texas within the Smith County. In Smith County, CC incidence has an age-adjusted rate of 7.5 per 100,000 [13,14]. In comparison, the CC incidence rate is 8.5 to 10.4 in Northeast Texas and 9.2 in overall Texas [13,14]. Mortality due to CC is 3.4 in Smith County; 3.5 in Northeast Texas and 2.8 in Texas [13,14]. In the present study, data outcome of a total of 173 women who presented for colposcopy at The University of Texas Health Science Center at Tyler from 2007 to 2017 were evaluated. All subjects provided initial informed consent for their examination and the data review was approved by the Institutional Review Board of The University of Texas Health Science Center at Tyler. All subjects are de-identified and only relevant information such as age, diagnosis, marital status, smoking, drinking history, and family history of cancer were used for statistical comparison. Family history of cancer was considered positive when parents, grandmother, grandfather, brother, sister or aunt was diagnosed with cancer. Past or present history of smoking (at least 1 pack/day) was considered positive. Similarly, past or present history of drinking alcohol (once a day) was considered positive. As no data were collected prospectively, a waiver of informed consent was granted by the Institutional IRB. During chart review, cases with substantial information available regarding the disease condition, age, marital status, repeated HPV/Pap testing, BMI and follow ups were considered informative.

### 2.2. Pap, HPV Testing and Colposcopic Examination

All the Pap tests were liquid-based either with ThinPrep or SurePath and were evaluated by relevant Pathologists using the 2001 Bethesda reporting system [15]. Human papilloma virus (HPV) testing on SurePath samples was conducted via Hybrid capture method and others were done using the Cobas HPV test. Colposcopy was performed using illumination and magnification after applying 5% acetic acid [16]. In addition to cervical biopsies, endocervical curettage was performed in certain clinical situations, including an unsatisfactory colposcopy following low-grade intraepithelial lesion, colposcopic evaluation of high-grade squamous intraepithelial lesion, and evaluation of all subcategories of atypical glandular cell cytology.

### 2.3. Follow-Up Treatment and Care 

Routine examination of cervical health and HPV infection status of these subjects was done by expert clinicians and the resident team at The University of Texas Health Science Center at Tyler. Biopsies were taken as indicated and evaluated by relevant pathologists. Appropriate treatment interventions were also employed as necessary per standard guidelines.

### 2.4. Hematoxylin and Eosin Staining of Tissue Biopsies

Biopsy tissue samples were fixed in formaldehyde and then paraffin embedded (FFPE) following standard protocol. Five micron sections were stained with the standard hematoxylin and eosin method and assessed by a histopathologist under a compound microscope. 

### 2.5. Statistical Analysis 

Statistical analysis was performed using IBM SPSS Statistics for Windows, Version 25.0 (IBM Corp., Armonk, NY, USA). Descriptive statistics were generated for all variables as reported in Table 1, Table 2 and Table 3. Pearson’s chi-square test was used to assess the distribution of those with HPV status, Pap test and colposcopy results across the various socio-demographic variables. Odds ratios (OR) and 95% confidence intervals (CI) were generated from binary logistic regression models to separately explore the likelihood that individuals with specific socio-demographic characteristics would have a particular HPV status, Pap test and colposcopy results.

## 3. Results

### 3.1. Patient Characteristics and Ethical Statement 

In this study, 173 women, aged 18–65 (mean 34.3) were examined in 2007–2017. In terms of medical coverage, 72% (123/171) of these women did not have any health insurance. For Ethnic distribution, out of the 166 informative cases, 102 (61%) were Caucasian (W), 34 (20%) were African-American (AA) and 32 (19%) were Hispanic/Latino (HIS). Of the informative cases, 34% (58/170) had normal BMI, 28.2% (48/170) overweight and 37.6% (64/170) obese. Of the 152 informative cases, 52 (34%) were married or living with a partner and 100 (66%) were unmarried, widowed or divorced. Of the 173 informative cases, 94 (54%) used contraception and 79 (46%) did not. The mean age of menarche of these subjects was 12.7 years and the mean number of births was 2.3. Of the 170 informative cases, 66 (39%) had a smoking history and 104 (61%) were non-smokers. Thirty percent (35/115) had a history of alcohol consumption, whereas 70% (80/115) did not have any history of alcohol consumption; 52% (55/105) had a family history of cancer and 48% (50/105) did not.

### 3.2. HPV, Pap testing and Colposcopic Outcomes

**Figure 1:**
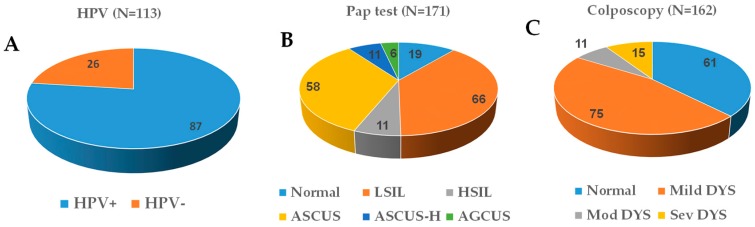
Data outcome of 173 cases that underwent human papilloma virus (HPV)/Pap testing and colposcopic examination. Individual pie charts indicate the frequency of women positive or negative for high-risk HPV infection (**A**), normal or abnormal Pap (**B**) and colposcopic (**C**) findings. N: total number of informative cases. LSIL: low-grade squamous intraepithelial lesion; HSIL: high-grade squamous intraepithelial lesion; ASCUS: atypical squamous cells of undetermined significance; ASCUS-H: atypical squamous cells, cannot exclude HSIL; AGCUS: atypical glandular cells of undetermined significance; Mod DYS: moderate dysplasia; Sev DYS: severe dysplasia.

The subjects were referred for colposcopy following either an abnormal Pap or positive hrHPV test. Colposcopy was done by a certified team of Physicians at the Family Medicine Clinic, The University of Texas Health Science Center at Tyler. Biopsies were carried out based on the abnormal cytologic findings and HPV-associated changes as determined by the team of Pathologists. Among the total 173 cases, follow-up data (at least 3 visits) were not available for 60 women and, as a result, they were regarded as non-informative. Of the 113 informative cases, 87 (77%) were positive for HPV and 26 (23%) were negative for any HPV infection (Figure 1A). An association between HPV positivity with young age (*p* = 0.002), and low number of pregnancies (*p* = 0.004) and births (*p* = 0.005) was observed (Table 1). Prolonged use of contraception (OR 1.93, 95% CI, 0.80–4.69) also appeared to increase the risk of HPV infection (Table 1). 

The Pap lesions were categorized as negative/normal, low-grade squamous intraepithelial lesion (LSIL), high-grade squamous intraepithelial lesion (HSIL), atypical squamous cells of undetermined significance (ASCUS), atypical squamous cells, cannot exclude HSIL (ASCUS-H) and atypical glandular cells of undetermined significance (AGCUS) using the Bethesda Nomenclature [10]. Of the 171 informative cases, 19 (11%) had a normal Pap test outcome, 66 (38%) had LSIL, 11 (6%) had HSIL, 58 (34%) had ASCUS, 11 (6%) had ASCUS-H and 6 (4%) had AGCUS (Figure 1B). When compared, unmarried/widowed/divorced women had an increased abnormal Pap test outcome compared to the married women or women living with a partner (*p* = 0.01) with over 278% increased odds (OR 3.78 at 95% CI, 1.29-11.10) (Table 2). Notably, African-American (OR 5.31, 95%CI, 0.67, 42.0) and Hispanic (OR 2.33, 95%CI, 0.50, 10.89) women also have a higher risk of abnormal Pap outcome compared to Caucasian American women. Alcohol consumption appeared to be protective (*p* = 0.04) for an abnormal Pap test outcome with about 67% reduced odds for those who reported the consumption of some form of alcohol compared to those who did not (OR 0.33, 95%CI, 0.11,0.99) (Table 2). A 77% increased odds of having an abnormal Pap outcome was observed among women with HPV infection compared to those who tested negative (OR 1.77, 95% CI, 0.48–6.44).

The colposcopy outcome was normal in 38% (61/162) of the cases, mild dysplasia was observed in 46% (75/162), moderate dysplasia in 7% (11/162) and severe dysplasia was evident in 9% (11/162) (Figure 1C). There was no statistically significant difference in the likelihood of abnormal colposcopic outcomes among the HPV-positive women compared to their negative counterparts (Table 3, OR 1.77, 95% CI, 0.69–4.56). However, there seemed to be a trend of higher likelihood (with about 77% increased odds) of having an abnormal colposcopic outcome among the HPV-positive women compared to their negative counterparts.

### 3.3. Follow-Ups and Interventions 

A total of 162 women received colposcopic examination. Moderate to severe dysplasia was detected in 12 subjects. A standard loop electrosurgical excision procedure (LEEP) was employed in these 12 subjects to prevent further progression of these lesions. One patient had progressed to invasive squamous cell carcinoma of the cervix and one subject was detected with grade I adenocarcinoma. One subject had undergone both LEEP followed by hysterectomy due to severe dysplasia.

## 4. Discussion

Cervical cancer develops and progresses through high-risk HPV infection and associated preneoplasitc changes in the uterine cervical epithelium. Importantly, timely screening and appropriate interventions can prevent progression of the preneoplasitc lesions to CC. Timely interventions through HPV/Pap testing and colposcopic evaluations are critical for early detection and prevention of CC, as demonstrated by other studies [3,17,18,19,20,21]. However, lack of adequate health insurance, race/ethnic distribution and poor socioeconomic status (SES), particularly in rural areas, could contribute considerably in CC preventive screening in a timely manner [15,16,17,18,19]. In the present study, we evaluated outcome data of women who received colposcopic evaluation from 2007 to 2017 at The University of Texas Health Science Center at Tyler to address this important aspect in rural East Texas. Notably, similar CC incidence rate combined with high mortality in Tyler compared to the State of Texas (13–14) suggest that a high-risk population inhabits this area. Our chart review data include insured and uninsured women with various racial and ethnic backgrounds. Multiple biopsies were taken followed by histopathological assessment and HPV testing to confirm preneoplastic epithelial changes and HPV infection in these women. Non-Hispanic African-American women had a higher risk of HPV infection, abnormal Pap and colposcopic outcome compared to the Hispanic and Caucasian American women, which is in accordance with other recent studies [21]. This outcome in African-American women could stem from multiple factors including socio-economical stress, environmental and biologic factors. Importantly, the functional relationship of the key biological/genetic factors associated with increased risk of CC in African-American women remains to be determined. Significantly higher incidences of HPV infection among young women with a low number of pregnancies and births suggest that they could be in a high-risk group for CC development. Sexual interaction at an early age is associated with a higher frequency of HPV infection [22]. Possibly as a reason, we have observed prevalent HPV infection and associated CC risk among younger women. Many of these young women, who are socio-economically stressed and living with multiple partners may be at greater risk for HPV infection/CC development. Although not significant, women who were married or living with partner exhibited an increased HPV infection rate compared to not married/widowed/divorced women, who may also be at low to moderate risk of CC development. Women with adequate medical coverage who received regular cervical examinations and repeat testing appeared to have a better outcome in terms of HPV infection management and CC development. In comparison, women with lack of adequate health coverage including African-American and Hispanic/Latina women had less frequent visits to the clinic for regular cervical examination, HPV/Pap testing and necessary colposcopic assessment, putting them at greater risk for CC development. This finding warrants regular screening of uninsured young women, including minorities, in rural East Texas, which may not only facilitate early detection of high-risk HPV infection but also prevent CC development. 

The study has limitations regarding the sample size. Tyler is an underserved rural area with an estimated population size of around 105,729 people [23]. Moreover, due to the lack of health literacy and related awareness, particularly among the uninsured minorities as demonstrated in other studies [24,25], limited visits could occur in the clinic for cervical health checkups. Another limitation is the lack of comprehensive follow-up data, which stemmed from the limited number of visits by the subjects. Currently, we are developing various awareness programs and expanding our questionnaire to accommodate more women and implement a better data collection plan through our Family Medicine and Women’s Wellness clinic at UHHSCT for a comprehensive survey and better management of CC in this area.

In summary, the present study identified a high frequency of relatively young women, including minorities, with hrHPV positivity and associated preneoplastic changes. Regular cervical health checkups in concert with the implementation of appropriate educational programs could be beneficial to encourage women to participate in CC screening programs, particularly in rural communities with low socioeconomic and weak educational backgrounds [26].

## Figures and Tables

**Table 1:** Association between HPV status and outcome from Pap test, colposcopic evaluation and various socio-demographic factors.

		HPV ^3^		OR(95%CI) ^4^	*p*-Value
Factor	Outcome	Positive N (%)	Negative N (%)		
**Race**	White	54 (76.1)	17 (23.9)	Ref	0.76
	Black	18 (81.8)	4 (18.2)	1.42 (0.42, 4.76)	
	Hispanic	13 (72.2)	5 (27.8)	0.82 (0.26,2.63)	
**Marital Status**	Married/Living with partner	29 (76.3)	9 (23.7)	1.18 (0.45, 3.04)	0.74
	Not Married/widowed/divorced	14 (20.9)	53 (79.1)	Ref	
**BMI ^1^**	Normal	26 (78.8)	7 (21.2)	Ref	0.94
	Overweight	25 (73.5)	9 (26.5)	0.96 (0.32, 2.90)	
	Obese	35 (79.5)	9 (20.5)	0.71 (0.25, 2.06)	0.53
**Insurance**	Yes	30 (88.2)	4 (11.8)	3.00 (0.95, 9.52)	0.05
	No	55 (71.5)	22 (28.6)	Ref	
**Contraception**	Yes	51 (82.3)	11 (17.7)	1.93 (0.80, 4.69)	0.14
	No	36 (70.6)	15 (29.4)		
**Family History of Cancer**	Yes	28 (77.8)	8 (22.2)	0.96 (0.33, 2.80)	0.93
	No	33 (78.6)	9 (21.4)		
**Smoking Status**	Yes	27 (69.2)	12 (30.8)	0.55 (0.23, 1.36)	0.19
	No	57 (80.3)	14 (19.7)		
**Alcohol Consumption**	Yes	17 (68.00)	8 (32.0)	0.60 (0.21, 1.70)	0.34
	No	46 (78.0)	13 (22.0)		
**Age**	Mean (standard deviation)	32.9 (11.1)	41.5 (12.0)	0.94 (0.91, 0.98)	0.002
**Number of Pregnancy**	Mean (standard deviation)	2.4 (1.8)	4.1 (2.7)	0.70 (0.55, 0.89)	0.004
**Number of Births**	Mean (standard deviation)	2.0 (1.6)	3.4 (2.2)	0.68 (0.52, 0.89)	0.005
**Age at Menarche**	Mean (standard deviation)	12.7 (1.4)	12.5 (1.8)	1.08 (0.59, 1.98)	0.81
**Pap ^2^**	Negative/normal	9 (69.2)	4 (30.8)	Ref	0.51
	LSIL	25 (78.1)	7 (21.9)	1.593 (0.37, 6.74)	
	HSIL	5 (83.3)	1 (16.7)	2.22 (0.19, 25.72)	
	ASCUS	44 (80.01)	11 (20.0)	1.78 (0.46, 6.86)	
	ASCUS-H	3 (50.0)	3 (50.0)	0.44 (0.06, 3.24)	
	AGCUS	0 (0.0)	0 (0.0)	-	
**Outcome of Colposcopy**	Normal	30 (73.2)	11 (26.8)	Ref	0.42
	Mild dysplasia	38 (80.9)	9 (19.1)	1.55 (0.57, 4.22)	
	Moderate dysplasia	7 (100.0)	0 (0.0)	-	
	Severe dysplasia	8 (80.0)	2 (20.0)	1.47 (0.27, 8.00)	

^1^ BMI: Body mass index was determined following National Heart Lung and Blood Institution criteria: Underweight = <18.5; Normal weight = 18.5–24.9; Overweight = 25–29.9; Obese = BMI of 30 or greater (0% was underweight). ^2^Pap: Papanicolaou test; ^3^HPV: Human papilloma virus, N = Number, ^4^OR = Odds Ratio, CI = Confidence Interval.

**Table 2:** Association between outcome from the Pap test and colposcopy, HPV status and socio-demographic factors.

		Pap Test ^3^		OR (95%CI) ^4^	*p*-Value
Factor	Outcome	Positive N (%)	Negative N (%)		
**Race**	White	87 (86.1)	14 (13.9)	Ref	0.14
	Black	33 (97.1)	1 (2.9)	5.31 (0.67, 42.0)	
	Hispanic	29 (93.5)	2 (6.5)	2.33 (0.50, 10.89)	
**Marital Status**	Married/Living with partner	41 (80.4)	10 (19.6)	Ref	**0.01**
	Not Married/widowed/Divorced	93 (93.9)	6 (6.1)	3.78 (1.29, 11.10)	
**BMI ^1^**	Normal	54 (94.7)	3 (5.3)	Ref	0.38
	Overweight	40 (83.3)	8 (16.7)	1.90 (0.45, 7.965)	
	Obese	57 (90.5)	6 (9.5)	0.53 (0.17, 1.63)	0.27
**Insurance**	Yes	44 (91.7)	4 (8.3)	1.32 (0.41, 4.28)	0.64
	No	108 (89.32)	13 (10.7)	Ref	
**Contraception**	Yes	86 (92.5)	7 (7.5)	1.81 (0.65, 5.00)	0.25
	No	68 (87.2)	10 (12.8)	Ref	
**Family History of Cancer**	Yes	45 (83.3)	9 (16.7)	0.56 (0.17, 1.79)	0.32
	No	45 (90.0)	5 (10.0)	Ref	
**Smoking Status**	Yes	59 (90.8)	6 (9.2)	1.18 (0.41, 3.35)	0.76
	No	92 (89.3)	11 (10.7)	Ref	
**Alcohol Consumption**	Yes	27 (77.1)	8 (22.9)	0.33 (0.11, 0.99)	0.04
	No	72 (91.1)	7 (8.9)	Ref	
**Age**	Mean (standard deviation)	34.0 (11.1)	37.1 (11.4)	0.98 (0.93, 1.02)	0.28
**Number of Pregnancy**	Mean (standard deviation)	2.8 (2.2)	2.3 (1.9)	1.14 (0.85, 1.540)	0.37
**Number of Births**	Mean (standard deviation)	2.3 (1.9)	2.2 (1.4)	1.04 (0.77, 1.41)	0.79
**Age at Menarche**	Mean (standard deviation)	12.7 (1.4)	12.1 (1.7)	1.42 (0.83, 2.44)	0.20
**HPV^2^**	Negative/normal	22 (84.6)	4 (15.4)	Ref	0.38
	Positive	78 (90.7)	8 (9.3)	1.77 (0.48, 6.44)	
**Outcome of Colposcopy**	Normal	53 (89.8)	6 (10.2)	Ref	0.63
	Mild dysplasia	67 (89.3)	8 (10.7)	0.95 (0.31, 2.90)	
	Moderate dysplasia	10 (90.9)	1 (9.1)	1.13 (0.12, 10.45)	
	Severe dysplasia	15 (100.0)	0 (0.0)	-	

^1^ BMI: Body mass index was determined following National Heart Lung and Blood Institution criteria: Underweight = <18.5; Normal weight = 18.5–24.9; Overweight = 25–29.9; Obese = BMI of 30 or greater (0% was underweight). ^2^ HPV: Human papilloma virus; ^3^ Pap test: Papanicolaou test, N = Number; ^4^ OR = Odds Ratio, CI = Confidence Interval.

**Table 3:** Association between outcome from colposcopy and HPV status, Pap test results and socio-demographic factors.

		Colposcopy		OR (95%CI) ^4^	*p*-Value
Factor	Outcome	Dysplasia N (%)	No Dysplasia N (%)		
**Race**	White	60 (63.2)	35 (36.8)	Ref	0.27
	Black	22 (66.7)	11 (33.32)	1.172 (0.51, 2.69)	
	Hispanic	14 (48.3)	15 (51.7)	0.54 (0.24, 1.26)	
**Marital Status**	Married/Living with partner	35 (71.4)	14 (28.6)	Ref	0.13
	Not Married/widowed/Divorced	55 (58.5)	39 (41.5)	0.56 (0.27, 1.19)	
**BMI^1^**	Normal	35 (66.0)	18 (34.8)	Ref	0.55
	Overweight	27 (60.0)	18 (40.0)	1.26 (0.59, 2.71)	
	Obese	37 (60.7)	24 (39.3)	0.97 (0.44, 2.14)	0.95
**Insurance**	Yes	28 (60.9)	18 (39.1)	Ref	0.87
	No	71 (62.3)	43 (37.7)	1.06 (0.53, 2.15)	
**Contraception**	Yes	55 (62.5)	33 (37.5)	1.01 (0.54, 1.92)	0.97
	No	46 (62.2)	28 (37.8)	Ref	
**Family History of Cancer**	Yes	38 (70.4)	16 (29.6)	1.58 (069, 3.65)	0.28
	No	27 (60.0)	18 (40.0)	Ref	
**Smoking Status**	Yes	37 (61.7)	23 (38.3)	0.97 (0.51, 1.91)	0.97
	No	62 (62.0)	38 (38.0)	Ref	
**Alcohol Consumption**	Yes	16 (48.5)	17 (51.5)	0.44 (0.19, 1.02)	0.05
	No	51 (68.0)	24 (32.0)	Ref	
**Age**	Mean (standard deviation)	33.5 (10.7)	35.0 (10.6)	0.99 (0.96, 1.02)	0.37
**Number of Pregnancy**	Mean (standard deviation)	3.0 (2.3)	2.4 (1.7)	1.16 (0.97, 1.39)	0.10
**Number of Births**	Mean (standard deviation)	2.5 (2.0)	2.1 (1.5)	1.13 (0.92, 1.37)	0.24
**Age at Menarche**	Mean (standard deviation)	12.6 (1.4)	12.7 (1.6)	0.96 (0.66, 1.39)	0.82
**HPV^2^**	Negative/normal	11 (50.0)	11 (50.0)	Ref	0.73
	Positive	53 (63.9)	30 (36.1)	1.77 (0.69, 4.56)	
**Pap^3^**	Normal	9 (56.3)	7 (43.8)	Ref	0.79
	LSIL	40 (63.5)	23 (36.5)	1.35 (0.44, 4.12)	
	HSIL	9 (81.8)	2 (18.2)	3.50 (0.57, 21.67)	
	ASCUS	33 (62.3)	20 (37.7)	1.28 (0.41, 3.99)	
	ASCUS-H	7 (63.6)	4 (36.4)	1.36 (0.28, 6.58)	
	AGCUS	3 (50.0)	3 (50.0)	0.78 (0.12, 5.10)	

^1^ BMI: Body mass index was determined following National Heart Lung and Blood Institution criteria: Underweight = <18.5; Normal weight = 18.5–24.9; Overweight = 25–29.9; Obese = BMI of 30 or greater (0% was underweight). ^2^ HPV: Human papilloma virus; ^3^ Pap: Papanicolaou test; ^4^ OR = Odds Ratio, CI = Confidence Interval.

## References

[1] Crosbie E.J., Einstein M.H., Franceschi S., Kitchener H.C. (2013). Human papilloma virus and cervical cancer. Lancet.

[2] Schiffman M., Solomon D. (2013). Clinical practice. Cervical-cancer screening with human papilloma virus and cytologic cotesting. N. Engl. J. Med..

[3] Texas Cancer Registry. http://www.dshs.texas.gov/tcr/.

[4] National Cancer Institute. https://www.cancer.gov/.

[5] Pratte M.A., Griffin A., Ogazi C., Yurasevecz S., Blanks C.A., McCooey L., Kaufman J.S. (2018). Racial/Ethnic Disparities in Cervical Cancer Screening Services Among Contractors of the Connecticut Breast and Cervical Cancer Early Detection Program. Health Equity.

[6] Su B., Qin W., Xue F., Wei X., Guan Q., Jiang W., Wang S., Xu M., Yu S. (2018). The relation of passive smoking with cervical cancer: A systematic review and meta-analysis. Medicine (Baltimore).

[7] Papillomavirus Episteme. https://pave.niaid.nih.gov/.

[8] Benard V.B., Castle P.E., Jenison S.A., Hunt W.C., Kim J.J., Cuzick J., Lee J.H., Du R., Robertson M., Norville S. (2017). New Mexico HPV Pap Registry Steering Committee. Population-Based Incidence Rates of Cervical Intraepithelial Neoplasia in the Human Papillomavirus Vaccine Era. JAMA Oncol..

[9] Kahn J.A. (2009). HPV vaccination for the prevention of cervical intraepithelial neoplasia. N. Engl. J. Med..

[10] Akinlotan M., Bolin J.N., Helduser J., Ojinnaka C., Lichorad A., McClellan D. (2017). Cervical Cancer Screening Barriers and Risk Factor Knowledge Among Uninsured Women. J. Community Health.

[11] Owusu G.A., Eve S.B., Cready C.M., Koelln K., Trevino F., Urrutia-Rojas X., Baumer J. (2005). Race and ethnic disparities in cervical cancer screening in a safety-net system. Matern Child Health J..

[12] Dockery L.E., Motwani A., Ding K., Doescher M., Dvorak J.D., Moore K.N., Holman L.L. (2018). Improving cancer care for American Indians with cervical cancer in the Indian Health Service (IHS) system—Navigation may not be enough. Gynecol. Oncol..

[13] Texas Cancer Registry (2018). Age-Adjusted Cancer Mortality Rates in Texas. https://www.cancer-rates.info/tx/.

[14] Texas Cancer Registry (2018). Age-adjusted Invasive Cancer Incidence Rates in Texas. https://www.dshs.state.tx.us›legislative›2018-Reports.

[15] Apgar B.S., Zoschnick L., Wright T.C. (2003). The 2001 Bethesda System terminology. Am. Fam. Physician.

[16] Apgar B.S., Kaufman A.J., Bettcher C., Parker-Featherstone E. (2013). Gynecologic procedures: Colposcopy, treatments for cervical intraepithelial neoplasia and endometrial assessment. Am. Fam. Physician.

[17] Wentzensen N., Walker J., Smith K., Gold M.A., Zuna R., Massad L.S., Liu A., Silver M.I., Dunn S.T., Schiffman M. (2018). A prospective study of risk-based colposcopy demonstrates improved detection of cervical precancers. Am. J. Obstet. Gynecol..

[18] Campbell C.M.P., Menezes L.J., Paskett E.D., Giuliano A.R. (2012). Prevention of invasive cervical cancer in the United States: Past, present, and future. Cancer Epidemiol. Biomarkers Prev..

[19] Kobetz E., Seay J., Amofah A., Pierre L., Bispo J.B., Trevil D.D., Gonzalez M., Poitevien M., Koru-Sengul T., Carrasquillo O. (2017). Mailed HPV self-sampling for cervical cancer screening among underserved minority women: Study protocol for a randomized controlled trial. Trials.

[20] Feldman S. (2011). Making sense of the new cervical-cancer screening guidelines. N. Engl. J. Med..

[21] Markt S.C., Tang T., Cronin A.M., Katz I.T., Howitt B.E., Horowitz N.S., Lee L.J., Wright A.A. (2018). Insurance status and cancer treatment mediate the association between race/ethnicity and cervical cancer survival. PLoS ONE.

[22] Louie K.S., de Sanjose S., Diaz M., Castellsagué X., Herrero R., Meijer C.J., Shah K., Franceschi S., Muñoz N., Bosch F.X. (2009). Early age at first sexual intercourse and early pregnancy are risk factors for cervical cancer in developing countries. Br. J. Cancer.

[23] QuickFacts. https://www.census.gov/quickfacts/tylercitytexas..

[24] Flores B.E., Acton G., Arevalo-Flechas L., Gill S., Mackert M. (2019). Health Literacy and Cervical Cancer Screening Among Mexican-American Women. Health Lit. Res. Pract..

[25] Webb S., Kelly P.J., Wickliffe J., Ault K., Ramaswamy M. (2019). Validating self-reported cervical cancer screening among women leaving jails. PLoS ONE.

[26] Musa J., Achenbach C.J., O’Dwyer L.C., Evans C.T., McHugh M., Hou L., Simon M.A., Murphy R.L., Jordan N. (2017). Effect of cervical cancer education and provider recommendation for screening on screening rates: A systematic review and meta-analysis. PLoS ONE.

